# Cell-Based and Cell-Derived Therapies Delivered During Ex Vivo Machine Perfusion for Solid Organ Reconditioning Prior to Transplantation: A Systematic Review

**DOI:** 10.3390/jcm15197698

**Published:** 2026-10-05

**Authors:** Mariam Alemi, Zara Edwards, Vassilios Papalois

**Affiliations:** 1 Imperial College London, South Kensington Campus, London SW7 2AZ, UK; zara.edwards21@imperial.ac.uk; 2 Department of Surgery and Cancer, Imperial College London, London SW7 5NH, UK; vassilios.papalois@nhs.net

**Keywords:** machine perfusion, normothermic machine perfusion, organ transplantation, cell therapy, cell-based therapy, mesenchymal stromal cells, extracellular vesicles

## Abstract

**Background/Objectives**: Organ shortage is a major barrier to transplantation, leading to the emergence of marginal donor organs and extended criteria donor grafts and, alongside these, machine perfusion as a platform for reconditioning grafts to meet transplantation standards. Despite functioning as a reconditioning platform in its own right, machine perfusion also allows for the delivery of therapeutic agents directly to grafts prior to transplant. Cell-based and cell-derived therapies have been increasingly explored for delivery during this window, although evidence is fragmented across individual organ systems, and whether ex vivo benefit is confirmed post-transplant remains unclear. This systematic review aimed to synthesise the evidence from both perfusion-phase findings and post-transplant findings across solid organ machine perfusion, evaluating the concordance between the two. **Methods**: This review was prospectively registered on PROSPERO and conducted in accordance with the Preferred Reporting Items for Systematic Reviews and Meta-Analyses (PRISMA) guidance. Included studies delivered cell-based or cell-derived therapies during ex vivo machine perfusion of a solid organ. The SYRCLE tool was used across all studies to assess risk of bias, and given substantial heterogeneity in cell type, protocol, and organ models, findings were synthesised narratively, with a sensitivity analysis restricted to the subset of studies incorporating a transplantation phase. **Results**: Thirty-one studies met inclusion criteria, spanning kidney (*n* = 10), liver (*n* = 11), lung (*n* = 9), and heart (*n* = 1). Mechanisms converged largely on immunomodulatory and metabolic pathways, most consistently reported across organs, alongside a less consistent but notable cross-organ parallel in regenerative progenitor activation between liver and kidney. Benefit was reported more consistently at the molecular and histological levels rather than the physiological level. Eight of the studies proceeded to transplantation, of which half showed a beneficial post-transplant result, and half showed mixed results. **Conclusions**: Cell-based and cell-derived therapies are best understood as adjuncts to the existing reconditioning capacity of machine perfusion. Molecular and mechanistic benefit was consistently reported; however, confirmation beyond the perfusion phase remained incomplete across organ systems. Mechanisms supported by direct interventional evidence represent priority candidates for further preclinical validation and independent replication.

## 1. Introduction

### 1.1. Background

Solid organ transplantation remains the definitive treatment for end-stage organ failure, but its clinical impact is fundamentally constrained by a persistent shortage of organs suitable for transplantation [1]. According to NHS Blood and Transplant’s most recent Organ and Tissue Donation and Transplantation Activity Report, 8096 patients were on the active UK transplant waiting list as of 31 March 2025—the highest number on record thus far [2]. Additionally, the average age of deceased donors is also rising—over-50s accounted for 63% of all deceased organ donors in 2024–2025 [2]. This ageing donor profile, alongside an increasing burden of donor comorbidity, compounds the shortfall faced by transplant centres in the United Kingdom.

In response, many centres have increasingly turned to extended criteria donor (ECD) and donation after circulatory death (DCD) organs to successfully expand the usable donor pool. The criteria defining an ECD organ vary by organ type: UK kidney allocation defines ECD kidneys as those from donors aged 60 years or older, or aged 50–59 years with at least two of hypertension, a terminal serum creatinine above 1.5 mg/dL, and death from cerebrovascular accident [3], while ECD livers are typically characterised by moderate-to-severe hepatic steatosis or other donor comorbidities that increase susceptibility to graft injury [4]. Importantly, these marginal grafts are inherently more vulnerable to ischaemia–reperfusion injury (IRI), and their historical use has been limited by poorer tolerance to prolonged cold ischaemic times under static cold storage (SCS), the standard preservation method for organs awaiting transplantation [5].

Recently, newer organ perfusion technologies, such as ex vivo normothermic machine perfusion (NMP), have emerged as a key strategy for overcoming some of the limitations associated with DCD and ECD organs [6]. NMP maintains organs at, or close to, body temperature whilst also perfusing them with nutrient- and oxygen-rich solutions, allowing organs to remain metabolically active ex vivo [7]. Beyond extending the safe preservation time, this metabolic activity allows for real-time assessment of organ viability and function prior to transplantation, a feature which static cold storage alone does not allow for [8]. Whilst the feasibility and safety of NMP has been demonstrated in multiple trials, supporting its clinical translation, there is still significant heterogeneity in the benefits of NMP alone between type of organ [9,10,11,12]. Furthermore, there is still debate as to whether NMP alone can adequately recover extremely vulnerable organs [12]. This has therefore motivated interest in using the perfusion period not merely to preserve the organs, but to actively modulate and recondition them prior to transplantation.

As the perfused organ is functionally isolated within a closed circuit, agents can be delivered directly to the graft in a controlled, organ-confined manner, avoiding the systemic distribution, metabolism, and clearance that would otherwise affect an intervention administered directly to the donor or recipient [12]. This principle has been explored across pharmacological, gene-based, and cell-based reconditioning strategies for liver, kidney and thoracic organs [13,14,15,16].

The use of cell-based therapies, particularly mesenchymal stromal cells (MSCs), has garnered particular interest [17]. MSCs have been investigated for their anti-inflammatory, immunomodulatory, and regenerative properties that make them an appealing candidate for mitigating IRI and supporting organ recovery during perfusion [17,18,19,20,21,22]. This approach has already progressed from preclinical models to early human application: a Newcastle-led study delivered multipotent adult progenitor cells (MAPCs) during human kidney NMP for the first time, reporting improved urine output, reduced expression of the injury biomarker NGAL, and favourable shifts in perfusate cytokine profiles [23]. These build on porcine work demonstrating that MSCs infused during kidney NMP can be traced to glomerular capillaries and are safe to administer prior to transplantation [24,25].

More recently, attention has extended from cell-based therapies to cell-derived, including extracellular vesicles (EVs). Both exosomes and MSC-derived microvesicles have been investigated as alternatives to MSCs. These vesicles are thought to mediate some of the paracrine signalling associated with the therapeutic effects of MSCs [26]. Furthermore, the use of cell-derived products may mitigate some of the risks and concerns regarding the use of live cells, including tumorigenicity, pulmonary entrapment, and immunogenicity [27]. EVs may also offer practical advantages, including easier storage and their ability to be frozen for prolonged periods of time [26]. To ensure consistent terminology, the classifications and definitions applied throughout this review are summarised in Table 1.

### 1.2. Rationale

There is an ever-growing body of preclinical evidence, particularly for EV-based strategies, supporting cell-based and cell-derived approaches to mitigating ischaemia–reperfusion injury across multiple solid organs; however, existing reviews have each captured only a partial picture of this extensive evidence base. Luijmes et al. produced a narrative mini-review spanning all four solid organs, but confined to whole-cell stem cell-based therapies, without a systematic search strategy, PROSPERO registration, or formal risk of bias assessment, and without extending to cell-derived acellular products [28]. Navez et al. conducted a systematic review and meta-analysis, but limited their scope to kidney transplantation only [29]. Most recently, Raza et al. systematically reviewed therapeutic interventions during machine perfusion but excluded lung entirely, narrowing their search to normothermic perfusion specifically. They identified no extracellular vesicle studies among their six included cell-based papers [15].

This review addresses these limitations by incorporating the four most commonly transplanted and preserved solid organs (kidney, liver, lung, and heart) [30], all modalities of ex vivo machine perfusion rather than normothermic perfusion alone, and both cell-based and cell-derived acellular therapies (including extracellular vesicles such as exosomes and mesenchymal stromal cell-derived microvesicles) within a single systematic framework. Additionally, we present a separate analysis restricted to only those studies with a re-transplantation phase, following cell administration during machine perfusion. To our knowledge, no prior systematic review has synthesised evidence across cell-based and cell-derived therapies, all four solid organ types, and the full range of machine perfusion modalities used for ex vivo organ reconditioning.

### 1.3. Objectives

We conducted a systematic review of cell-based and cell-derived therapies administered during ex vivo machine perfusion of liver, kidney, lung, and heart grafts prior to transplantation. Our primary objectives were to: (1) characterise the range of cell-based (e.g., mesenchymal stromal cells) and cell-derived acellular therapies (e.g., extracellular vesicles, including exosomes and MSC-derived microvesicles) reported, alongside the study models and perfusion protocols in which they were delivered; (2) synthesise reported effects on graft injury, viability, and function across these therapy classes; and (3) evaluate the methodological quality and risk of bias of the included evidence, including the extent to which preclinical findings have progressed toward human ex vivo and clinical validation, including studies with a transplantation phase following machine perfusion. This review aims to inform the design of future interventional perfusion studies and to characterise the current translational evidence for cell-based and cell-derived approaches to organ reconditioning.

### 1.4. Terminology and Intervention Classification

For the purposes of this review, interventions were classified into three classes: cell-based therapies, cell-derived acellular products, and organoid-based therapies. Progenitor-based therapies involving intact progenitor cells were classified as a subtype of cell-based therapy.

## 2. Materials and Methods

### 2.1. Protocol and Registration

This review was conducted in accordance with the PRISMA 2020 guidelines [27]. The protocol was registered prospectively on PROSPERO (CRD420261358704), on 3 April 2026. PRISMA 2020 Checklist is provided as Supplementary Materials (File S1).

### 2.2. Eligibility Criteria

Eligible studies delivered a cell-based or cell-derived therapy directly during ex vivo machine perfusion of a solid donor organ (kidney, liver, lung, or heart) in the context of organ preservation or reconditioning. Eligible therapies were MSCs, multipotent adult progenitor cells (MAPCs), MSC-derived EVs, organoids, or organ-specific progenitor cells. All cell sources, doses, delivery routes, and perfusion modalities were eligible. Comparators were standard-perfusate controls, including machine-perfused organs receiving vehicle without cell-based or cell-derived supplementation, and contralateral-organ or within-donor controls. Eligible outcomes included measures of organ viability, IRI, and cell behaviour during perfusion, with post-transplant functional, feasibility, and safety outcomes also eligible where studies extended to transplantation.

Studies were excluded if therapies were delivered in vivo rather than ex vivo; if the model was limited to in vitro cell or organoid culture without intact organ perfusion; if the intervention was pharmacological or gene-based; or if machine perfusion was not used as the delivery platform. Restrictions were not placed on species, sex, age, or weight. Only English-language full-text publications were eligible, with no restriction on publication date.

### 2.3. Search Strategy

MEDLINE (via PubMed), Embase, and Web of Science were searched from database inception to 6 April 2026, with no restriction on publication date. Searches were limited to English-language publications. Full search strategies are provided in the Supplementary Material (File S2).

### 2.4. Study Screening

**Figure 1:**
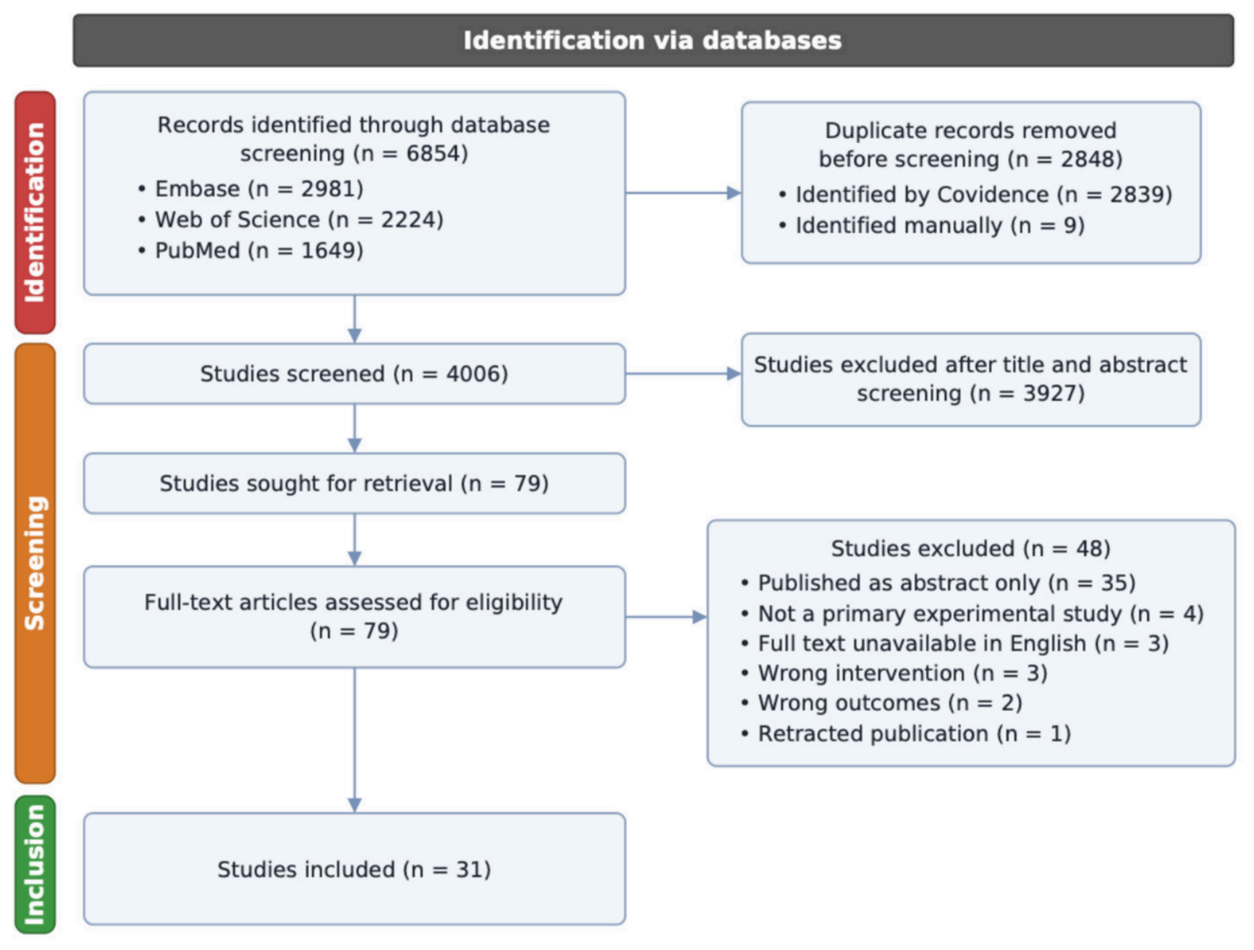
The PRISMA 2020 flow diagram of study identification, screening, and inclusion. Thirty-one studies were included in the final review.

The search results were imported into Covidence, which removed duplicates. Two reviewers independently screened titles and abstracts against the pre-specified eligibility criteria. Full texts of potentially eligible articles were similarly assessed independently by both reviewers. Discrepancies at either stage were resolved by group discussion between the reviewers. Study selection is summarised in the PRISMA 2020 flow diagram (Figure 1).

### 2.5. Data Extraction

Data were extracted independently by two reviewers using a piloted Covidence extraction form. Extracted domains included study identification and design, donor/population characteristics, intervention and comparator details, and outcome data (dichotomous, continuous, or qualitative as appropriate). Comparator groups were categorised according to the nature of the comparator, including machine perfusion alone, standard preservation, vehicle/sham or non-treated controls, alternative therapeutic interventions, paired or within-organ controls, and no separate comparator. Study-specific comparator details are reported in Table 2. Conflicts between reviewers were resolved by discussion and consensus.

### 2.6. SYRCLE Risk of Bias Assessment

Risk of bias was assessed independently by two reviewers using SYRCLE’s risk of bias tool. In accordance with the protocol, the NIH Study Quality Assessment Tool was planned for human organ studies, while SYRCLE was pre-specified for animal studies. During the review process, we considered the NIH tool less applicable to the included human studies, which were experimental ex vivo studies of discarded organs rather than studies involving human participants. We therefore used a modified application of the SYRCLE framework to provide a consistent, structured assessment of experimental risk of bias. For human ex vivo studies, animal-specific domains that were not applicable to isolated human organs were recorded as not applicable, with remaining domains assessed where sufficient information was available. This constituted a deviation from the protocol and is specifically acknowledged in the manuscript. As SYRCLE was developed for animal studies and has not been validated specifically for human ex vivo organ perfusion models, its application to these studies represents an adapted framework. Disagreements were resolved by discussion between reviewers. 

### 2.7. Data Synthesis

Meta-analysis was not performed due to anticipated heterogeneity in organ and cell type, perfusion modality, and outcome reporting. Findings were synthesised narratively and grouped by organ type and cell-based therapy category, with subgroup patterns by organ, therapy and perfusion modality described rather than statistically pooled. A sensitivity analysis excluding feasibility/proof-of-concept studies lacking a separate control group assessed whether conclusions held when restricted to controlled studies.

## 3. Results

### 3.1. Study Selection

Searching identified 6854 records across three databases (Embase, n = 2981; Web of Science, n = 2224; PubMed, n = 1649). Following deduplication (n = 2848 removed), 4006 records were screened by title and abstract, of which 3927 were excluded. Full-text screening included 79 records, of which 48 were excluded, most commonly due to being published as abstract only (n = 35). Thirty-one studies met the eligibility criteria and were included in the review. The study selection process is summarised in Figure 1.

### 3.2. Study Characteristics

**Figure 2:**
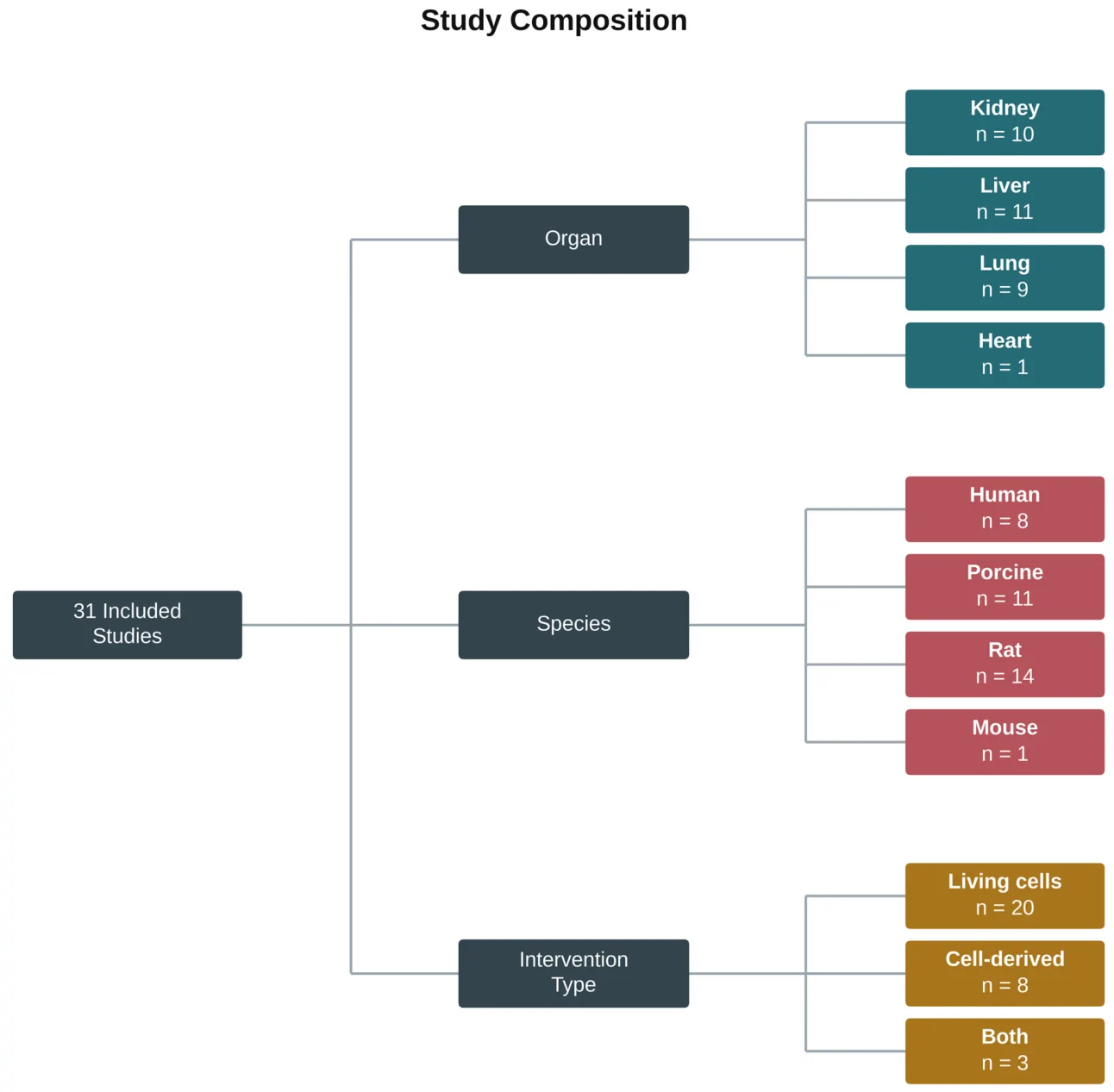
Study composition by organ, donor species, and intervention type (n = 31 included studies).

Among the 31 included studies, the liver was the most frequently studied organ (n = 11), followed by kidney (n = 10), lung (n = 9), and heart (n = 1). Twenty studies delivered living cell therapy, eight delivered cell-derived acellular products, and three evaluated both within the same study; two studies evaluated organoid-based therapies. Donor organs were most commonly rat (n = 14) or porcine (n = 11), with human organs declined for transplantation used in eight studies and a single murine model; species counts exceed 31 as some studies involved more than one species. Study distribution across organ, species, and intervention type is summarised in Figure 2. Therapies were most often delivered by vascular infusion (arterial, portal venous, or pulmonary arterial) or perfusate supplementation, with a smaller number using airway, biliary or bioreactor-mediated routes. Most studies used controlled or randomised preclinical designs, of which eight extended to transplantation with assessment of post-transplant outcomes. Comparator structures varied across studies, including machine perfusion alone, standard preservation, vehicle/sham or non-treated controls, alternative therapeutic interventions, and paired or within-organ controls. Most studies had a defined comparator, although two studies included study arms without a separate comparator. Study characteristics are summarised in Table 2.

### 3.3. Risk of Bias

**Figure 3:**
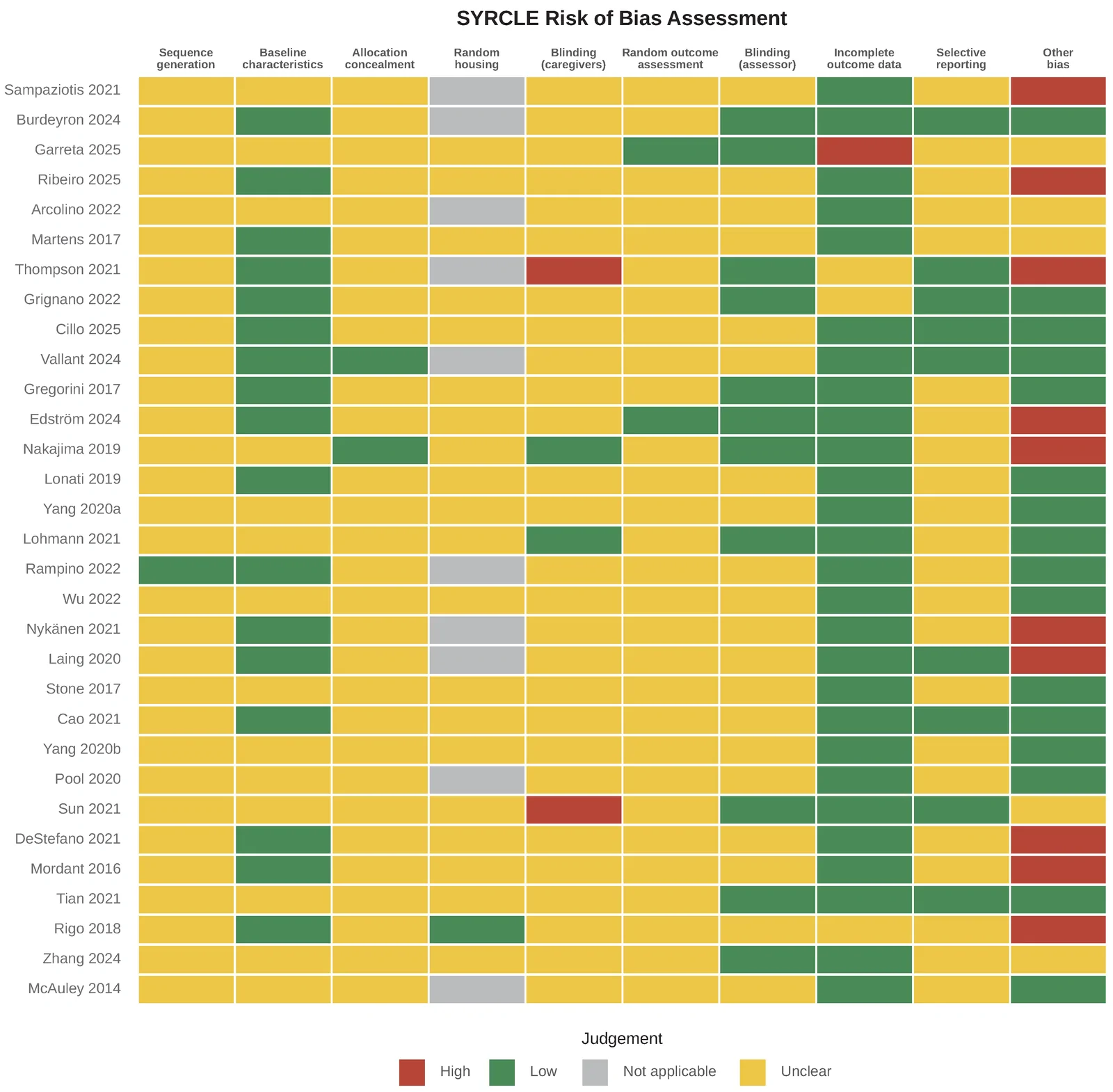
A risk of bias assessment across all 31 included studies, using an adapted version of SYRCLE’s risk of bias tool. Judgements are shown as low, unclear, high, or not applicable for each domain [1,12,17,22,23,24,31,32,33,34,35,36,37,38,39,40,41,42,43,44,45,46,47,48,49,50,51,52,53,54,55].

The risk of bias judgments are presented as a colour-coded risk of bias matrix generated in R (Version 4.4.1) (Figure 3). Randomisation-related domains were poorly reported throughout: sequence generation was unclear in 30 of the 31 studies and allocation concealment unclear in 29, with unclear outcome assessment also in 29 studies. Random housing was not applicable in 10 studies using human or abattoir-sourced organs. Blinding was inconsistently reported: blinding of caregivers was rated high risk in two studies and low risk in two, remaining unclear elsewhere. Blinding of outcome assessors, however, was low risk in 11 studies.

Outcome-related domains performed considerably better, with incomplete outcome data rated low risk in 27 studies, baseline characteristics in 16 studies, and selective reporting in nine. Other sources of bias was the most polarised domain, rated high risk in 10 studies, most commonly reflecting financial or intellectual-property interests by authors in the therapy being tested. Overall, judgements were largely unclear rather than high risk, in line with incomplete methodological reporting rather than flaws in study conduct.

### 3.4. Effect Direction by Outcome Domain

**Figure 4:**
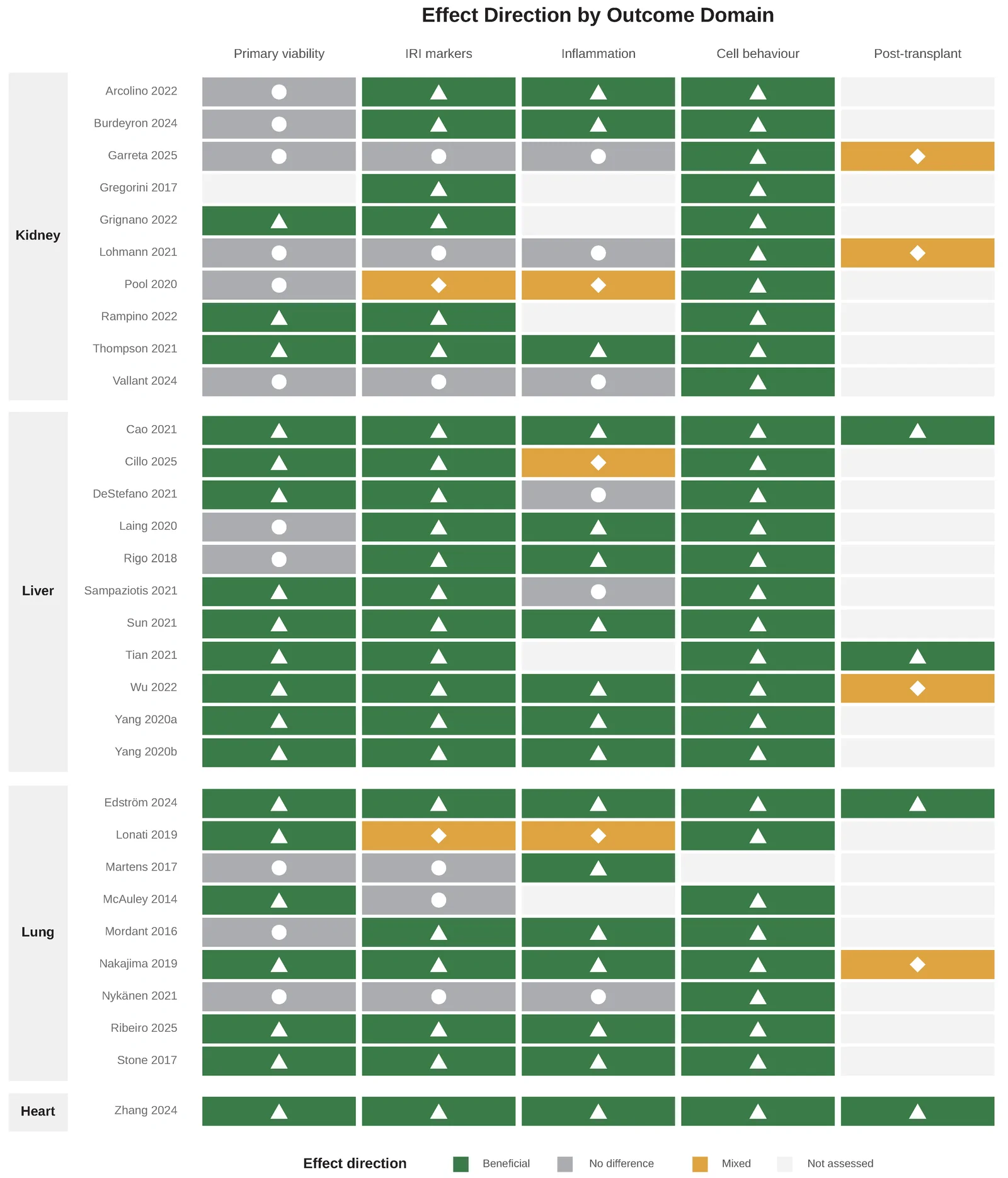
Effect direction by outcome domain across all 31 included studies, grouped by organ. The cells indicate whether the reported effect was beneficial (statistically significant improvement), mixed, no different from control, or not assessed [1,12,17,22,23,24,31,32,33,34,35,36,37,38,39,40,41,42,43,44,45,46,47,48,49,50,51,52,53,54,55].

Reported effect directions across outcome domains are summarised in Figure 4. Consistently assessed domains were primary viability, IRI markers, and inflammation, evaluated in most included studies, while post-transplant outcomes were assessed in only eight. Within assessed domains, statistically significant benefit was the predominant direction of effect, with improvements in injury markers and cell behaviour reported across kidney, liver, and lung studies. Null findings clustered in kidney studies, where several reported no significant difference from control. Inflammation was the most heterogeneous domain: two studies reported mixed effects—significant benefit on some markers alongside significant increases in pro-inflammatory chemokines [34,47]. By contrast, of the eight studies assessing post-transplant outcome, four reported a beneficial result [24,32,37,42,43,46,51,55], and four reported a mixed result [24,32,44,52]. Effects were therefore most favourable in mechanistic domains, while transplant-linked outcomes showed a less complete pattern of confirmed benefit, although this was only assessed in a small proportion of studies.

### 3.5. Narrative Synthesis

#### 3.5.1. Kidney

Ten studies assessed cell-based or cell-derived therapies during kidney perfusion across rat, porcine and human declined donor models. Delivery was predominantly intra-arterial and feasibility was demonstrated across all studies.

Injury biomarkers (perfusate or urinary LDH, AST, and NGAL) improved significantly in half of the studies, spanning both living cell and vesicle-based products [1,23,31,34,35]. Histological corroboration was less consistent: three studies reported structural improvement—reduced damage scores [17], better preserved mitochondrial ultrastructure [35] and reduced tubular dilatation [1]—while two studies found no histological difference from control [25,33].

Mechanistic findings clustered around immune, metabolic, and regenerative pathways. Immunomodulatory effects were reported by Arcolino et al. and Thompson et al. Arcolino et al. showed reduced IL-1β, TNF-α, IL-2 and IL-8, alongside increased IDO (via the kynurenine:tryptophan ratio) [31]; Thompson et al. showed reduced IL-1β and increased IL-10 and IDO activity, with Thompson additionally demonstrating suppressed neutrophil chemotaxis functionally [23]. By contrast, Vallant et al. reported a non-significant trend towards higher IL-1β with MSC treatment, the only study in this dataset showing this direction of effect [36]. The most direct causal evidence came from Grignano et al., who showed that the protective effect of EVs on tubular proliferation was abolished by CD73 silencing, implicating CD73–adenosinergic signalling in ATP restoration (*p* < 0.0001) [33]. Gregorini et al. separately demonstrated metabolic restoration via upregulated respiratory electron transport and citric acid cycle genes [17]. Regenerative activation was a unique finding in Arcolino et al., in which de novo SIX2 expression, a foetal nephron progenitor marker, was reported in over 40% of proliferating proximal tubular cells versus none in controls [31].

Physiological function improved less consistently than molecular markers. Thompson et al. reported significantly increased urine output (*p* = 0.009) and improved microvascular perfusion, although they did not assess creatinine clearance and fractional sodium excretion [23]. Where these were assessed elsewhere, results were mixed: Pool [34], Arcolino [31] and Vallant [36] showed no significant improvement in creatinine clearance, while Lohmann et al. found no MSC-specific benefit in GFR but a significant reduction in plasma creatinine with human MSCs on days 4–7 post-transplant (*p* ≤ 0.032) [24]. Pool et al. reported mixed effects, with reduced injury markers alongside significantly increased IL-6 and IL-8 [34].

A transplantation phase was performed in two independent studies [24,32], though only one included a functional-benefit comparison. Garreta et al. confirmed organoid engraftment and persistence at 24–48 h post-transplant, with radiance increasing over this window, indicating cell viability [32]. Lohmann et al. reported one graft loss during the 14-day porcine auto-transplant period (NMP + vehicle group, day 6, delayed graft function), with no improvement in GFR attributable to MSCs [24]. Evidence of the therapeutic benefit in kidneys therefore remains largely confined to the ex vivo perfusion window and is unconfirmed beyond the perfusion phase.

#### 3.5.2. Liver

Eleven publications reported cell-based or cell-derived therapies during liver perfusion across rat, porcine and human discarded-donor models [12,22,37,38,39,40,41,42,43,44,45]. Delivery was predominantly portal venous, with one indirect/paracrine bioreactor model [38] and one intraductal delivery route for a structural, non-IRI indication [40]. Importantly, six of the eleven included liver studies originate from this single laboratory [37,41,42,43,44,45]. As such, more than half of the whole-cell BMMSC evidence base in this section should be read as one coherent but mechanistically heterogeneous line of evidence requiring external replication, not as independent confirmations of the same effect.

Injury biomarkers improved consistently with whole-cell BMMSC therapy across the rat DCD studies: ALT, AST and cumulative bile production improved significantly with BMMSC co-perfusion relative to NMP alone in both papers by Yang et al. [44,45] and Sun et al. [41]; lactate also improved significantly in both studies by Yang et al., but not in that by Sun et al. When measured longitudinally post-transplant, the same pattern held in three further papers [37,42,43]. Evidence for EV-based products was more mixed: DeStefano et al. reported significant ALT/AST reduction with HLSC-EV treatment at both doses tested [12], whereas Rigo et al., using the same EV product from the same source laboratory, found no significant between-group difference in ALT and only a single timepoint of significance for LDH, despite being at a comparable low EV dose [39]. This discrepancy may reflect differences in injury model (isolated hypoxic perfusion vs. a DCD warm-ischaemia model) rather than treatment failure, but it means EV efficacy for standard biochemical injury markers cannot be considered consistently demonstrated across the two available EV studies.

Histological corroboration followed a similar pattern. Suzuki injury scores improved significantly with BMMSC treatment in both papers by Yang et al. and Sun et al. [41,44,45] (e.g., Sun 2021: 1.6 ± 0.55 vs. 3.4 ± 0.55 in NMP alone). DeStefano et al. reported histological improvement (necrosis score) only at the higher EV dose [12], while Rigo et al. reported a significantly improved Suzuki score with EV treatment (3.9 ± 0.4 (EV-treated) vs. 5.7 ± 0.6 (control), *p* = 0.030) [39].

Mechanistic findings clustered around three pathways. First, oxidative stress and mitochondrial protection were demonstrated most extensively in the Yang et al. cohort, with BMMSC co-perfusion improving mitochondrial AST, JC-1 membrane potential ratio, GSH, MDA and MPO relative to NMP alone [44,45]; a parallel ferroptosis-based mechanism was demonstrated by Sun et al. via significant GPX4 upregulation (*p* = 0.004 vs. NMP, *p* < 0.001 vs. SCS) [41]. Second, immunomodulatory and microcirculatory pathways were consistently reported: the same Yang 2020 cohort showed BMMSC-associated improvement across the full endothelial/adhesion marker panel (eNOS, iNOS, ET-1, ICAM-1, VCAM-1, von Willebrand factor, nitric oxide, PAF, thrombomodulin), while Wu and Cao et al. demonstrated reduced graft cytokines (TNF-α, IFN-γ) and inflammatory chemokine expression, attributing this to dendritic cell maturation suppression and NKT co-inhibitory receptor regulation respectively, with HO-1-modified BMMSCs consistently outperforming unmodified BMMSCs [37,43]. Third, a distinct regenerative-activation pathway was reported by Tian et al., in which HO-1-BMMSC treatment upregulated peribiliary gland progenitor markers (SOX9, Nanog, CFTR) and tight-junction proteins (occludin, ZO-1) via Wnt/β-catenin signalling [42]—a finding conceptually paralleled by the foetal nephron progenitor activation reported in the kidney synthesis [31], suggesting endogenous regenerative programme activation may be a cross-organ phenomenon rather than kidney-specific. By contrast, DeStefano 2021’s broad inflammatory RT-PCR panel showed no significant differences between any group [12].

Structural and functional regeneration was most directly demonstrated by Sampaziotis et al., where organoid-derived cells regenerated an estimated 40–85% of injected bile ducts, providing the clearest evidence across the entire liver dataset of durable structural engraftment rather than transient biomarker modulation [40]. Cillo et al.’s. indirect bioreactor/secretome paradigm produced more equivocal results: bile production improved significantly in the rat model but not the porcine model, and several markers (liver oedema index, hepatic energy charge, NAD +/NADH ratio) were not statistically significant despite favourable trends, suggesting paracrine-only exposure may be a mechanistically distinct and currently less robust delivery strategy than direct intra-organ cell or EV injection [38]. Laing et al.’s feasibility outcome (perfusate lactate viability criterion, met in three of six livers) was not reported against the internal split-lobe control statistically, so this study contributes proof-of-concept delivery/distribution data rather than an efficacy signal [22].

Post-transplant survival data were available from three studies conducted by the same Tianjin research group using a shared rat DCD orthotopic liver transplant platform; two of these included an allogeneic rejection model with an FK506 comparator [37,43], while the third assessed bile duct/ischaemic injury instead [42]. In the two rejection-model studies, survival showed a consistent gradient—SCS/NMP alone shortest, BMMSC (BP) intermediate, HO-1-modified BMMSC (HBP) longest and statistically indistinguishable from FK506 on survival specifically, though not on rejection severity [37,43] (Wu et al. reported significantly worse rejection activity index vs. FK506, *p* < 0.05).

#### 3.5.3. Lung

Nine studies assessed cell-based or cell-derived therapies during lung machine perfusion, across murine, porcine and human discarded-donor models, using whole MSCs/MAPCs [48,49,50,52], lung-derived exosomes [53], a direct head-to-head MSC-versus-EV comparison within a single study [54], MSC-derived EVs in an isolated ex vivo model [47], and two further whole-MSC studies incorporating a transplantation phase [46,51].

Physiological and functional outcomes showed the clearest organ-specific pattern in this dataset: a recurring dissociation between molecular/inflammatory improvement and physiological benefit. Martens et al. found no significant difference between MAPC and control for any physiological parameter (oxygenation, compliance, pulmonary vascular resistance, wet/dry weight ratio, all *p* ≥ 0.13), despite significant reductions in several inflammatory markers in the same study (BAL neutrophils *p* = 0.02; IFN-γ, IL-1β and TNF-α in BAL fluid all *p* = 0.002) [48]. Mordant et al. showed an almost identical pattern: no significant functional benefit across the full dose range tested, with significance reached only in a two-group comparison at the highest dose (150 × 10^6^ MSCs IV: compliance *p* = 0.043, PaO_2_ *p* = 0.005), while perfusate IL-8 suppression reached significance independently of physiological outcomes, and VEGF was significantly upregulated relative to baseline (*p* = 0.008) though not relative to control (*p* = 0.095) [50]. Ribeiro et al. showed a related but distinct pattern: robust, significant improvement across oxygenation, compliance, airway pressure and vascular resistance, but only when lung-derived exosomes were administered pre-DCD (before warm ischaemic injury was established); post-DCD (rescue) administration still produced significant improvement in airway pressure (*p* = 0.003) and compliance (*p* = 0.01), but lung injury score (*p* = 0.09) and TUNEL (*p* = 0.1) did not reach significance, indicating that the timing of delivery relative to injury affects histological but not all physiological outcomes [53]. By contrast, McAuley et al. demonstrated a clear positive functional signal on its primary endpoint, with MSC treatment significantly restoring alveolar fluid clearance relative to perfusion-only controls (*p* < 0.001), although perfusate oxygenation and pulmonary artery pressure were unchanged in the same study [49]. Nakajima et al. showed the same dissociation pattern in a transplant-phase model with significant EVLP-phase improvement in HGF, apoptosis markers, and several cytokines, but only peak airway pressure among the physiological measures (*p* = 0.04) [51].

Histological and injury-marker findings were more consistently favourable than functional outcomes where whole-cell or EV products were delivered before or alongside injury. In a mouse model of donor lung reconditioning, Stone et al. did not directly compare MSCs and MSC-derived EVs statistically, but both significantly reduced pulmonary oedema and neutrophil infiltrations relative to Steen solution alone [54]. Ribeiro et al.’s pre-DCD exosome group similarly showed significant reductions in lung injury score and apoptosis (TUNEL scoring) relative to control, again with the post-DCD group showing attenuated, non-significant effects on these two markers specifically [53]. Martens et al.’s histological injury scores (congestion, necrosis, neutrophil influx) showed no significant difference for MAPC versus control, consistent with that study’s broader pattern of molecular-without-physiological benefit [48].

Mechanistic findings pointed to two distinct pathways, one confirmed pharmacodynamically and one demonstrated by antibody blockade. Nykänen et al. confirmed successful pulmonary delivery of a genetically engineered IL-10-secreting MSC product, with perfusate IL-10 approximately 100-fold higher and lung tissue transgenic IL-10 mRNA roughly 15-fold higher than endogenous levels within the same treated lungs; the study also assessed downstream physiological and injury outcomes (PVR, oxygenation, compliance, airway pressure, wet/dry ratio), finding no significant treatment effect on any of these despite confirmed transgene expression, and its own title states this delivery was ‘compromised by acidic lung microenvironment,’ implying that confirmed transgene expression did not translate into the expected therapeutic effect [52]. McAuley et al. provided more direct causal mechanistic evidence: co-administration of a KGF-neutralising antibody significantly abolished the MSC-mediated improvement in alveolar fluid clearance (*p* < 0.01), directly implicating keratinocyte growth factor signalling as the mediating mechanism [49]. Mordant et al. additionally established a clear dose–response relationship for pulmonary MSC retention (*p* = 0.022); intravenous delivery reached significance on its own comparisons while intrabronchial delivery did not, though no direct statistical test between the two routes was reported [50]. Edstrom et al., a transplant-phase study, reported proteomic downregulation of neutrophil-recruitment and NET-formation pathways, despite non-significant HGF (*p* = 0.829) [46], unlike Nakajima et al.’s significant EVLP-phase finding, possibly reflecting distinct MSC sources (umbilical cord versus term amniotic-fluid-derived) [51].

Lonati et al. delivered MSC-derived EVs rather than whole cells or lung-derived exosomes and reported reduced PVR (*p* = 0.040), increased NO metabolites (*p* = 0.027) along with restored tissue ATP (*p* = 0.009) [47]. Notably, leukocytes were reduced in the perfusate but increased in tissue alongside elevated hyaluronan, suggesting redistribution rather than suppression—a mechanistic pattern distinct from the whole-cell studies above.

Two studies proceeded to transplantation. Nakajima et al. found significant post-transplant reductions in tissue TNF-α, oedema, and histological injury (all *p* ≤ 0.03), without physiological improvement [51]. Edstrom et al. showed the clearest transplant-linked benefit: zero PGD versus significantly higher rates in controls (*p* = 0.0041), with improved oxygenation and compliance (*p* = 0.0043 each) [46]. Transplant-confirmed benefit in the lung is therefore limited but not entirely absent.

#### 3.5.4. Other

One study, Zhang 2024, delivered human umbilical cord MSC-derived exosomes during ex vivo perfusion of a rat DCD heart, followed by heterotopic transplantation and 90 min of in vivo reperfusion [55]. Exosome treatment reduced myocardial injury, with lower histopathological injury scores, fewer TUNEL-positive cardiomyocytes, and suppression of pro-inflammatory cytokines IL-1β, IL-18 and TNF-α. Transcriptomic evidence implicated the integrin/PI3K/Akt survival pathway and downregulation of the pro-apoptotic gene Bad. Post-transplant mechanical function (left ventricular developed pressure and dP/dt) was also significantly improved relative to vehicle across multiple balloon volumes—eight of nine sub-comparisons reached significance, with dP/dt max not reaching significance at the highest volume tested—a more consistent functional signal than during the ex vivo heart perfusion phase itself, where significance was reached at only one tested volume [55]. This study represents the only independent cell-derived therapy study in a heart perfusion context to date.

### 3.6. Sensitivity Analysis

Of the 31 studies included, eight incorporated a transplantation phase and were used to evaluate whether ex vivo perfusion-phase findings persist beyond the perfusion window [24,32,37,42,43,46,51,55] (Table 3).

Post-transplant outcomes were mixed in four of the eight studies. In kidney, both transplant studies showed mixed results: Lohmann et al. found no significant MSC-specific benefit in GFR, though plasma creatinine was significantly lower with human MSCs specifically on days 4–7 post-transplant (*p* ≤ 0.032), with no equivalent effect in the porcine-MSC arm [24]. Garreta et al. confirmed organoid engraftment at 24–48 h post-transplant, but immune and inflammatory cell markers (CD3+, CD4+, CD8+, CD68+, CD15+, CD45+) showed no significant difference from control [32]. In the liver, Wu et al. also showed mixed results: survival was statistically indistinguishable from FK506, but rejection activity index was significantly worse than FK506 despite this survival parity, indicating that survival and rejection activity can dissociate even within a single concordant study [43]. Similarly, Nakajima et al. showed mixed results in the lung: molecular and histological outcomes improved significantly post-transplant, but physiological function (PaO_2_/FiO_2_, compliance, peak airway pressure) did not, echoing the patterns seen ex vivo [51].

Four studies showed direct beneficial post-transplant results. Cao et al. and Tian et al., from the same Tianjin research group, both reported significantly prolonged survival with HO-1-modified BMMSCs; in Cao et al., this was statistically indistinguishable from the FK506 comparator, though rejection severity in unmodified BMMSC-treated animals remained intermediate between NMP alone and the HO-1-modified arm [37,42]. Tian et al. lacked an FK506 or SCS comparator arm, limiting the calibration of effect size against standard alternatives. Zhang et al. reported significantly improved post-transplant mechanical function across eight of nine sub-comparisons. Left ventricular developed pressure and dP/dt min were significant at all three tested balloon volumes, while dP/dt max was significant at two of the three tested volumes—based on a 90 min follow-up window, the shortest in this subset [55]. Edstrom et al. showed the clearest results in the dataset, with zero primary graft dysfunction events versus significantly higher rates in controls, alongside significantly improved post-transplant oxygenation and compliance [46].

**Figure 5:**
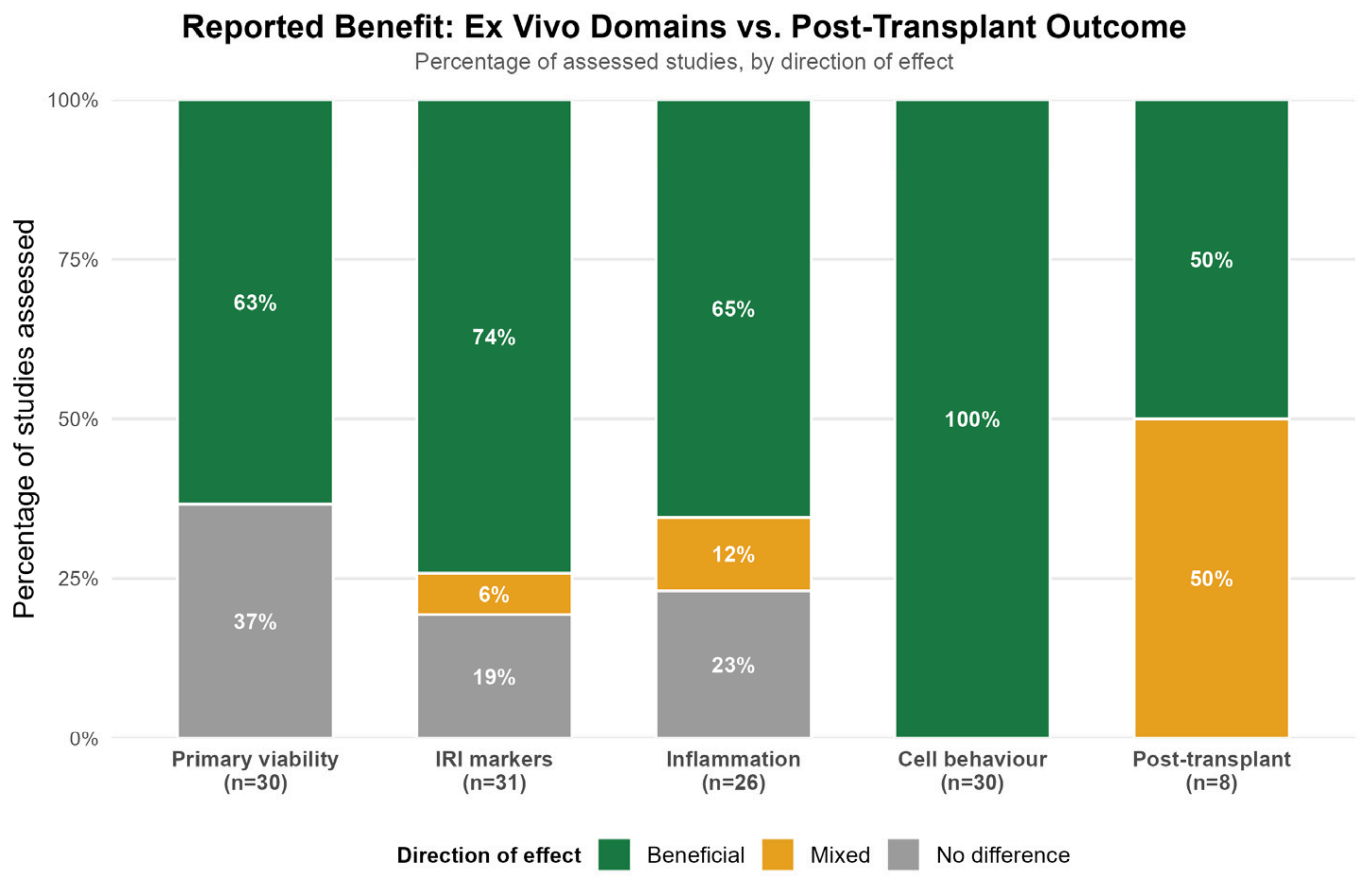
The reported benefit of interventions across ex vivo and post-transplant outcome domains. The percentage of assessed studies reporting beneficial, mixed, or no difference outcomes for each of five domains (primary viability, IRI markers, inflammation, cell behaviour, and post-transplant outcomes). Studies in which a given outcome was not assessed were excluded from that domain’s denominator.

Overall, of the eight studies proceeding to a transplantation phase, four were classified as beneficial, while four showed mixed results. None of the studies reported worse outcomes in treated groups. This was a consistent pattern across organs with more than one transplant-phase study—both kidney studies had mixed results, and one of the two liver studies was also mixed. Post-transplant benefit in this dataset is evident but incomplete rather than a simple confirmed/unconfirmed split. Treated groups consistently outperformed controls on at least one outcome, but seldom across every outcome tested, and the measures falling short varied unpredictably from study to study. Ex vivo perfusion biomarkers therefore appear to be, at best, partial predictors of post-transplant benefit across this dataset (Figure 5).

## 4. Discussion

### 4.1. Summary

Machine perfusion, independent of additional therapeutic agents, functions as a reconditioning platform in its own right—organs discarded for transplant have been salvaged and transplanted successfully following NMP rehabilitation across kidney, lung, liver, and heart [56,57,58,59]. The interventions catalogued in this review should therefore be interpreted as adjuncts to this existing rehabilitative capacity rather than as free-standing therapies tested in isolation.

Three patterns emerged across the 31 studies spanning the four organs reviewed. First, despite a broad therapeutic landscape, mechanisms essentially converged into immunomodulatory, metabolic, and regenerative pathways. Notably, the parallel between regenerative progenitor activation in both the liver and kidney (peribiliary gland progenitors via Wnt/β-catenin signalling and SIX2+ nephron progenitors respectively) may reflect a shared injury response rather than purely organ-specific effects, based on a single study per organ, and should be interpreted with caveats (Section 4.3). Second, benefits were reported far more consistently at the molecular and histological level rather than at a physiological function level, a discordance observed frequently in the lung dataset but also echoed elsewhere. Third and most consequentially, only eight of the 31 studies proceed to a transplantation phase, highlighting that molecular/histological improvement was rarely tested against a transplant endpoint. A sensitivity analysis restricted to these eight studies found that post-transplant confirmation was incomplete rather than a simple confirmed/unconfirmed split: while 63–74% of studies reported molecular or histological benefit, only half of the eight transplant-phase studies showed an uncomplicated post-transplant benefit, with the remainder showing a mixed picture of partial success that recurred across every organ with more than one transplant-phase study, rather than clustering in a single organ.

Studies also differed in whether the organ tested was human or animal, and separately, in whether the cellular therapy was human-derived or matched to the organ’s species—two dimensions that are easy to conflate. The most clinically direct evidence came from studies using human organs and human-derived interventions [22,23,31,35,40,49,52], none of which proceeded to transplantation. By contrast, studies reaching a transplantation phase were split between same-species animal models and xenogeneic models delivering human-derived cells, EVs, or organoids into an animal recipient. Lohmann et al. tested both same-species and human-derived MSC arms within the same porcine kidneys [24]. No study in this review combined a human organ, a human-derived product, and confirmed post-transplant outcomes. The mechanistic basis for these patterns is considered in more detail below.

A related imbalance concerns organ representation in the field and compounds the translational gap identified in the sensitivity analysis. The majority of included studies were accounted for by kidney (n = 10) and liver (n = 11), with lung following closely (n = 9), while the heart was represented by a single study. This likely reflects genuine logistical and biological barriers associated with cardiac machine perfusion, which carries technical demands not shared by the other organs in this review—separate management of preload and afterload, and a mandatory cardioplegic phase at both initiation and termination of perfusion [60]. Functional assessment is similarly underdeveloped: the only FDA-approved clinical device preserving the heart in a non-working state lacks the ability to generate functional data, meaning clinicians rely on visual inspection and circulating lactate, both of which are substandard predictors of post-transplant outcome [61]. Where these barriers have been overcome, as in the current dataset, the resulting evidence has been compelling—an encouraging signal that this gap is one of research capacity rather than a ceiling of what cardiac machine perfusion can achieve.

### 4.2. The Translational Gap

The consistent yet incomplete post-transplant benefit noted in the sensitivity analysis above raises an interpretive question about whether this is a biological, methodological or artefactual pattern.

Two findings in the dataset represent a genuinely biological explanation whereby an intervention may act on some pathophysiological processes and not others—consistent with this review’s mechanistic findings above, where the strongest-evidenced pathways (CD73–adenosinergic signalling, keratinocyte growth factor signalling) were single-target, narrow mechanisms rather than broad acting ones. Wu et al.’s dissociation between prolonged survival and incompletely suppressed rejection severity reflects this pattern: an intervention producing a partial rather than absent effect, plausibly because HO-1-modified BMMSCs treatment effects are confined to one part of the alloimmune response, leading to rejection activity being only partially controlled [43]. Similarly, Lohmann et al.’s significant reduction in plasma creatinine without a corresponding change in GFR illustrates how closely related outcomes can diverge—a pattern equally consistent with a genuine biological effect or with statistical under-powering [24].

A second possibility is methodological and draws from a limitation identified in the risk of bias assessment—group sizes across transplant-phase studies ranged from eight animals per arm at the upper end to as few as just one animal in some comparisons. A ‘null’ result drawn from a single animal therefore carries essentially no statistical power, and even the modestly larger samples elsewhere (typically n = 5–8 animals per arm) make it difficult to distinguish a true absence of effect from an under-powered one.

A related, artefactual concern is financial: around 30% of studies across the full dataset disclosed a commercial or intellectual-property tie to the product’s manufacturer—a conflict-of-interest pattern independently associated with more favourable reporting in the wider literature [62]. This does not establish biassed reporting within the dataset but is a limitation on the interpretability of those studies’ findings, that a simple increase in sample size would not be able to resolve.

### 4.3. Proposed Mechanisms of Action

The precise biological mechanisms by which cell-based and cell-derived therapies confer benefit during machine perfusion remain incompletely understood, and the strength of mechanistic evidence varies considerably across included studies. The majority of mechanistic observations in this review are associative, describing molecular, cellular or histological changes alongside an apparent therapeutic effect, rather than directly demonstrating a specific pathway’s involvement. Only two studies provided direct interventional evidence linking pathway modulation to therapeutic benefit; one implicating CD72-adenosinergic signalling in the kidney and one involving KGF signalling in the lung [33,50]. This therefore represents the strongest causal evidence currently available in the included literature.

Growing evidence in the wider literature favours a paracrine rather than engraftment-based model of action: therapeutic cells are frequently cleared rapidly from the perfusion circuit, and benefit is instead thought to arise from secreted bioactive molecules including cytokines, growth factors, and extracellular vesicles carrying regulatory proteins and microRNAs, rather than sustained integration into recipient tissue [29]. This is consistent with the pattern observed across this dataset, where EVs reproduced immunomodulatory and metabolic effects comparable to whole-cell products; one study directly demonstrated statistically indistinguishable benefit between MSCs and MSC-derived EVs within the same experiment [54]. Whilst these findings support a potential paracrine contribution to therapeutic activity, they do not establish paracrine signalling as the sole mechanism of action.

Immunomodulation is the most frequently reported proposed mechanism for reducing ischaemia–reperfusion injury during perfusion, generally attributed to a shift in the local cytokine balance—suppression of pro-inflammatory mediators alongside upregulation of anti-inflammatory and tolerogenic signals—rather than direct immune cell elimination [29]. This pattern recurred across all three solid organs assessed for immune outcomes in the present review, with reduced pro-inflammatory cytokines and increased tolerogenic markers reported in kidney, liver, and lung studies alike. The recurrence of these observations across organs suggests that immunomodulatory responses may be a common feature of the interventions studied; however, the evidence is predominantly associative and does not establish that these changes are causally responsible for the observed improvements in graft outcomes. Notably, this convergence was not absolute: one kidney study reported a trend in the opposite direction, underscoring that immunomodulatory response is not a uniform or guaranteed feature of these therapies.

A second, closely related proposed mechanism is metabolic and mitochondrial protection. Here, it is proposed that cellular or vesicular therapy supports recipient cell bioenergetics directly, either by supplying substrates, protecting mitochondrial integrity, or activating protective metabolic pathways such as adenosine signalling or antioxidant defence [33]. Evidence for this mechanism spanned all three organs in the present dataset, including preserved oxidative phosphorylation gene expression, restored gluconeogenic capacity, protected mitochondrial membrane potential, and a distinct ferroptosis-suppression pathway. This suggests metabolic support may be a broadly shared feature of these interventions rather than an organ-specific curiosity.

A third, less frequently reported mechanism is the reactivation of endogenous developmental progenitor programmes following graft injury—an independently well-established phenomenon in each organ. In the liver, peribiliary glands house SOX9+ progenitor cells that differentiate into mature cholangiocytes in response to bile duct injury, via Wnt-mediated proliferation and Notch-mediated maturation [63,64]. These are directly implicated in post-transplant biliary stricture formation [65]. In the kidney, SIX2+ progenitor cells are exhausted by 36 weeks of gestation and had never been observed to re-express in adult human kidney tissue prior to findings from Arcolino et al. [31].

The present dataset provides an early signal that these pathways can be reactivated in the context of therapeutic machine perfusion: HO-1-modified BMMSC treatment in the liver upregulated SOX9/Nanog/CFTR markers characterising activation-to-maturation trajectory, while nKSPC-based therapy in the kidney induced de novo SIX2 expression alongside SOX9 and VEGF [31,37]. Although these observations provide an intriguing parallel between liver and kidney, each organ is represented by only one study in the present dataset. The findings should therefore be regarded as preliminary and hypothesis-generating rather than evidence of a conserved cross-organ mechanism.

Overall, the mechanistic findings in this review cluster around immunomodulatory, metabolic and regenerative responses; yet, the consistency and evidential strength of these pathways differ significantly. Immunomodulatory and metabolic/mitochondrial effects were reported across multiple studies and organs but were predominantly associative, whereas regenerative/progenitor activation was supported by more limited evidence and remains preliminary. By contrast, direct interventional evidence was identified for only CD73–adenosinergic signalling in the kidney [33] and KGF signalling in the lung [49].

### 4.4. Risk of Bias Implications

Overall, this dataset generally had strong reporting standards for data completeness (87% low risk) but weak standards for describing randomisation and allocation (all >90% unclear)—largely a reporting gap rather than poor conduct since various studies claim randomisation without a named protocol [23,36,41,42] whilst others describe no allocation process at all [17,34,39,48].

A protocol deviation should be noted: although the NIH Study Quality Assessment Tool was originally specified for human studies, this was considered less applicable to the included human studies, which were ex vivo experiments involving perfusion of donor organs rather than studies involving human participants. An adapted version of the SYRCLE framework was therefore applied across all 31 studies in this review, rather than switching to alternative tools for the human tissue subset. This decision was deliberate to maintain consistency across the risk of bias assessment in a very mixed dataset. However, this produced some unconventional results, most notable in Domain 4 (random housing), which assumes live-animal custody and cannot be meaningfully applied to ex vivo human studies. Such studies were classified as not applicable accordingly, with remaining domains assessed where sufficient information was available. Much of the not applicable/unclear ratings on this domain likely reflects the modified application rather than a genuine unaddressed risk.

A subset of studies disclosed financial or intellectual-property ties to the manufacturer of tested products [22,23,39]. This was notably absent in the Tianjin liver cluster of six related studies [37,41,42,43,44,45], which reported no commercial conflicts but were instead funded through overlapping national grants. This limitation is therefore institutional rather than financial and should not be conflated. It remains unclear whether control groups across these six studies were independently generated or drawn from a shared baseline cohort, suggesting a further layer of potential non-independence beyond the noted authorship overlap. Finally, a number of studies used small or uneven sample sizes, as low as n = 3–4, which should warrant caution when interpreting null findings reported in this review.

### 4.5. Strengths and Limitations

This systematic review has several strengths. To our knowledge, it is the first review to comprehensively evaluate the effects of cell-based and cell-derived therapies delivered during ex vivo machine perfusion for solid organ reconditioning prior to transplantation, across the four most transplanted organs: kidney, liver, lung, and heart. The review was prospectively registered on PROSPERO and conducted in accordance with PRISMA guidance, with risk of bias formally assessed for all included studies using the SYRCLE tool in Covidence, lending methodological rigour to the synthesis. Additionally, cross-organ coverage allowed for a broad evaluation of cell-based and cell-derived therapies across the field, rather than a narrower single-organ focus. Given substantial heterogeneity in cell type, perfusion protocol, and organ model across included studies, narrative synthesis was adopted in preference to meta-analysis; this was a deliberate methodological choice rather than a limitation. Likewise, the eight-study transplant-phase sensitivity analysis was intentionally restricted to structured narrative comparison rather than subgroup meta-analysis, given comparable heterogeneity across species, follow-up duration, and outcomes.

However, this review also has limitations. The evidence base was dominated by animal and ex vivo models, with limited clinical translation data, highlighting a gap between preclinical mechanistic findings and their applicability to human transplantation. Additionally, a subset of included studies demonstrated institutional non-independence, raising the possibility that the literature in the field has arisen from overlapping research groups rather than independent authors. A related concern was financial, whereby approximately 30% of included studies disclosed a commercial or intellectual-property tie to the manufacturer of the product being tested: a pattern independently associated with more favourable reporting in the wider literature [62]. Furthermore, sample sizes were often small, particularly among the transplant-phase studies which ranged from eight animals per arm to as few as a single animal in some post-transplant comparisons. This limits the ability to draw conclusions from null findings, since they cannot be reliably distinguished from under-powered ones. Finally, outcome reporting was inconsistent across the studies: some presented results only in graphical form without extractable numerical values, which limited the completeness of data extraction and comparability across studies.

### 4.6. Future Research and Clinical Translation

Advancing the evidence base requires specifically designed studies that address uncertainties raised in this discussion. Comprehensive, null-inclusive outcome reporting would allow for genuine biological effects—even partial ones—to be separated from false negatives, alongside prospectively calculated sample sizes to clarify whether an absence of effect reflects a true null or just lack of statistical power, particularly for secondary functional and immunological endpoints. Handling of conflict-of-interest status as a pre-specified analytic variable would help disentangle genuine effects from reporting- or funding -related artefacts, supporting more reliable judgements about the underlying evidence.

The mismatch is most evident in the kidney, which carries the strongest mechanistic evidence in this review yet both kidney transplant-phase studies showed only a mixed post-transplant result. Grignano et al.’s demonstration that CD73 silencing abolished the therapeutic effect of extracellular vesicles is the clearest causal evidence across all organs but is confined to the ex vivo phase [33]. Independent replication of these results alongside comprehensive post-transplant outcomes evaluated with adequate power should be a priority before this mechanism is considered for early clinical evaluation.

Cardiac research faces a different priority. The single cardiac study included produced one of the strongest results in this review, but this rests on a single study in a field where technical demands and the paucity of reliable functional assessment tools limit research [60,61]. Investing in standardised, working-mode functional endpoints is a prerequisite for evaluating the heart on comparable terms to other organs.

The evidence base is yet to support clinical adoption of any intervention discussed in this review; the translational gap makes it clear that even the strongest mechanistic findings cannot be assumed to confer benefit in a transplanted human recipient. It does, however, support an order of priority, whereby mechanisms with direct, interventional evidence (i.e., CD73–adenosinergic signalling in kidney and keratinocyte growth factor signalling in lung) are the strongest candidates for further preclinical validation and independent replication before clinical translation can be considered. For now, these interventions remain promising adjuncts to an already-effective platform, not yet independently proven therapies in their own right—the clearest priority for research seeking to expand the pool of viable donor organs.

## Figures and Tables

**Table 1:** Definitions of cell-based, cell-derived, and organoid-based therapies as used in this review.

Classification	Term	Definition
Cell-based therapy	Cell-based therapy	Use of intact, viable cells during ex vivo machine perfusion to support tissue repair, regeneration, or organ recovery. Examples include mesenchymal stromal cells (MSCs) and multipotent adult progenitor cells (MAPCs).
	Mesenchymal stromal cells (MSCs)	A class of stromal cells isolated from tissues such as bone marrow or adipose tissue. Can be used as a whole-cell therapy, as well as a source of cell-derived therapies.
	Progenitor-based therapy	Use of progenitor cells or progenitor-derived cellular populations as a means of tissue repair/regeneration.
Cell-derived acellular product	Cell-derived product	An acellular product obtained from cells that contains no intact viable cells, including extracellular vesicles and cell-derived secretomes.
	Extracellular vesicles (EVs)	A broad umbrella term for lipid bilayer-enclosed particles released by cells that cannot replicate. Distinct subtypes include exosomes and microvesicles.
Organoid-based therapy	Organoid	A three-dimensional cellular structure generated from stem or progenitor cells that recapitulates selected structural and/or functional features of a tissue and may support tissue repair/regeneration.

**Table 2:** Characteristics of included studies (n = 31).

Organ	Study	Species/Model	Injury Model	Cell/Product Type	Route	Design (n)	Comparator	Key Outcomes Measured	Key Findings
Kidney	Arcolino 2022 [31]	Human	DCD	Cell-based (progenitor nKSPC)	Vascular (arterial)	Controlled ex vivo study, n = 6 (3 vs. 3)	NMP alone	Primary viability; IRI markers; inflammation; cell behaviour	Reduced injury markers and inflammatory cytokines; de novo SIX2 re-expression in proximal tubular cells
	Burdeyron 2024 [1]	Porcine	Slaughterhouse-sourced, 32 min WIT	Cell-derived (UPC-EVs)	Perfusate/circuit	Randomised preclinical, n = 17 (3 arms: G1 n = 5, G2 n = 6, G3 n = 6)	Vehicle (PBS)	Primary viability; IRI markers; inflammation; cell behaviour	Reduced injury markers (LDH, ASAT) and pro-inflammatory cytokines; benefit confined to dual HMP + NMP dosing arm (G3)
	Garreta 2025 [32]	Porcine/Human (xeno)	Living-donor auto-transplant/slaughterhouse-sourced	Organoid-based (hPSC-derived organoids)	Vascular (arterial)	Preclinical, n = 7 (auto-transplant) + n = 8 (NMP-only)	Mock (no organoid infusion); NMP-only controls	Primary viability; IRI markers; inflammation; cell behaviour; post-transplant	No significant difference vs. mock in IRI or inflammatory markers; organoid engraftment confirmed
	Gregorini 2017 [17]	Rat	DCD	Cell-based + cell-derived (MSC and EV)	Perfusate/circuit	Controlled preclinical, n = 15 (3 arms) + n = 5 non-perfused	Belzer Solution (BS) only	IRI markers; cell behaviour	Reduced histological signs of ischemic injury; improved metabolic gene expression with MSC/EV treatment
	Grignano 2022 [33]	Rat/ Human (xeno)	DCD	Cell-based + cell-derived (MSC and EV)	Perfusate/circuit	Randomised preclinical, n = 15 (4 arms)	BS alone/CD73-silenced EVs	Primary viability; IRI markers; cell behaviour	Reduced renal damage score and improved cell proliferation with MSC/EV treatment; protective effects abolished in CD73 silencing implicating CD73–adenosinergic axis
	Lohmann 2021 [24]	Porcine	Surgically induced warm ischaemia, 75 min WIT	Cell-based (porcine and human MSC)	Vascular (arterial)	Randomised preclinical auto-transplant study, n = 28 (4 arms, n = 7 each)	oxHMP only/NMP + vehicle	Primary viability; IRI markers; inflammation; cell behaviour; post-transplant	No MSC-specific benefit on function, injury, or survival beyond oxHMP effect alone
	Pool 2020 [34]	Porcine/Human (xeno)	Slaughterhouse-sourced, 20 min WIT	Cell-based (BM-MSC/A-MSC)	Vascular (arterial)	Controlled preclinical ex vivo study, n = 20 (4 arms, n = 5 each)	NMP alone; circuit-only control	Primary viability; IRI markers; inflammation; cell behaviour	Reduced injury markers but paradoxically increased IL-6/IL-8; marker-specific effects/non-uniform
	Rampino 2022 [35]	Human	DBD, ECD (Remuzzi score >= 5)	Cell-derived (MSC-EVs)	Perfusate/Vascular (arterial)	Pilot randomised clinical study, n = 10 (5 pairs)	HOPE alone	Primary viability; IRI markers; cell behaviour	Reduced global renal damage score with better preserved ultrastructure
	Thompson 2021 [23]	Human	DCD/DBD declined donor kidneys	Cell-based (MAPC)	Vascular (arterial)	Paired randomised ex vivo study, n = 10 (5 pairs)	Contralateral paired kidney + crystalloid vehicle	Primary viability; IRI markers; inflammation; cell behaviour	Improved microvascular perfusion and urine output; reduced NGAL; cytokine profile shifted towards anti-inflammatory state
	Vallant 2024 [36]	Rat/ Porcine/Human	Rat: living donor; pig: abattoir-sourced; human: DCD/DBD declined	Cell-based (MSC)	Perfusate/Vascular (arterial)	Preclinical, n = 18 total	HMP alone (porcine); no comparator for rat/human arms	Primary viability; IRI markers; inflammation; cell behaviour	Feasibility demonstrated; successful glomerular and tubular engraftment without compromising physiological function
Liver	Cao 2021 [37]	Rat	DCD, 30 min WIT	Cell-based (BMMSC +/− HO-1)	Vascular (portal vein)	Controlled preclinical allogeneic study, n = 108 (6 arms)	NMP alone; SCS; NMP +unmodified BMMSC; NMP + FK506; Sham	Primary viability; IRI markers; inflammation; cell behaviour; post-transplant	Improved liver function, reduced injury/inflammation, and longer survival with BMMSC +/− HO-1
	Cillo 2025 [38]	Rat/ Porcine/Human (xeno)	Rat: standard procurement; pig: DCD-type (1h WIT)	Cell-derived (MSC-bioreactor secretome)	Bioreactor/indirect	Randomised preclinical, n = 26 (rat n = 20 + pig n = 6)	NMP alone	Primary viability; IRI markers; inflammation; cell behaviour	Improved bile production and reduced LDH; effects confined to rat cohort
	DeStefano 2021 [12]	Rat/ Human (xeno)	DCD-simulated	Cell-derived (HLSC-EV)	Perfusate/circuit	Randomised preclinical ex vivo study, n = 24 (4 arms, dose–response)	Warm ischemia (WI) + vehicle; non-ischaemic control	Primary viability; IRI markers; inflammation; cell behaviour	Dose-dependent reduction in injury markers; no effect on inflammatory gene panel
	Laing 2020 [22]	Human	Declined ECD livers (DBD/DCD)	Cell-based (MAPC)	Vascular (hepatic artery/portal vein)	Pilot feasibility human ex vivo study, n = 6	Within-liver/contralateral lobe control	Primary viability; IRI markers; inflammation; cell behaviour	Single-arm feasibility study; proportion of livers meeting viability criteria reported descriptively
	Rigo 2018 [39]	Rat/ Human (xeno)	Hypoxic injury	Cell-derived (HLSC-EV)	Perfusate/circuit	Controlled preclinical ex vivo study, n = 19 (2 arms)	NMP alone	Primary viability; IRI markers; inflammation; cell behaviour	HLSC-EVs internalise rapidly by hepatocytes; reduces hypoxic tissue injury, apoptosis and expression of hypoxia-induced genes
	Sampaziotis 2021 [40]	Human	Deceased donor liver, ischemic duct injury	Organoid-based (cholangiocyte organoids)	Duct/ biliary	Pilot human ex vivo study, n = 3 livers	Carrier-injected control ducts; untreated/control ducts	Primary viability; IRI markers; cell behaviour	Feasibility study; engraftment and functional repair of the biliary epithelium
	Sun 2021 [41]	Rat	DCD, 30 min WIT	Cell-based (BMMSC)	Vascular (portal vein)	Controlled preclinical ex vivo study, n = 24	NMP alone; SCS	Primary viability; IRI markers; inflammation; cell behaviour	Improved viability markers; consistent selective benefit of BMMSCs
	Tian 2021 [42]	Rat	DCD, 30 min WIT	Cell-based (BMMSC +/− HO-1)	Vascular (portal vein)	Controlled preclinical transplant study, n = 20–24 (5 arms)	NMP alone; Sham; NMP + wild-type BMMSCs (BP); XAV-939 treated HBP	Primary viability; IRI markers; cell behaviour; post-transplant	Improved survival, reduced apoptosis, and enhanced regenerative markers with HO-1-modified BMMSCs
	Wu 2022 [43]	Rat	DCD, cardiac arrest induction, 30 min WIT	Cell-based (BMMSC, HO-1-modified)	Vascular (portal vein)	Controlled preclinical transplant study, n = 36 (survival cohort)	Sham; NMP alone; SCS; NMP + BP; NMP + FK506	Primary viability; IRI markers; inflammation; cell behaviour; post-transplant	Markedly prolonged survival and reduced injury with HO-1-modified BMMSC treatment
	Yang 2020a [44]	Rat	DCD, surgical cardiac death, 30 min WIT	Cell-based (BMMSC)	Vascular (portal vein)	Controlled preclinical ex vivo study, n = 50	NMP alone; SCS	Primary viability; IRI markers; inflammation; cell behaviour	Improved liver enzymes, reduced oxidative stress and mitochondrial injury with BMMSC treatment
	Yang 2020b [45]	Rat	DCD, surgical cardiac death, 30 min WIT	Cell-based (BMMSC)	Vascular (portal vein)	Controlled preclinical ex vivo study, n = 50	NMP alone; SCS	Primary viability; IRI markers; inflammation; cell behaviour	Improved endothelial function markers and reduced microcirculatory injury with BMMSC treatment
Lung	Edström 2024 [46]	Porcine/Human (xeno)	LPS-induced ARDS injury model	Cell-based (TAF-MSC)	Mixed/ multi-route	Controlled preclinical transplant study, n = 22 (11 pairs)	EVLP alone	Primary viability; inflammation; cell behaviour; post-transplant	Improved physiological compliance and gas exchange; profoundly suppressed inflammation and prevented severed PGD
	Lonati 2019 [47]	Rat/ Human (xeno)	Euthanasia model, 15 min cold ischemia	Cell-derived (EVs)	Perfusate/circuit	Controlled preclinical ex vivo study, 3 groups n = 5	Untreated IR control; native non-perfused control	Primary viability; IRI markers; inflammation; cell behaviour	Restored ATP and reduced vascular resistance; some inflammatory/oxidative markers increased (adaptive response)
	Martens 2017 [48]	Porcine/Human (xeno)	DCD, 90 min WIT	Cell-based (MAPC)	Airway	Controlled preclinical ex vivo study, n = 12	Albumin-plasmalyte vehicle	Primary viability; IRI markers; inflammation	Reduced pro-inflammatory cytokines and neutrophil infiltration; no physiological improvement
	McAuley 2014 [49]	Human	Declined donor lungs (injury/comorbidity)	Cell-based (MSC)	Perfusate/circuit	Controlled human ex vivo study, n = 14 (4 arms)	Perfusion only	Primary viability; IRI markers; cell behaviour	Restored alveolar fluid clearance (KGF-dependent mechanism); no change in physiological parameters
	Mordant 2016 [50]	Porcine	18 h cold ischemia injury	Cell-based (HUCPVC/MSC)	Airway (IB)/Vascular (IV)	Controlled preclinical ex vivo study, n = 19 (5 arms)	No-cell control	Primary viability; IRI markers; inflammation; cell behaviour	Reduced IL-8 and improved VEGF with MSC treatment; no physiological improvement
	Nakajima 2019 [51]	Porcine/Human (xeno)	24 h cold preservation-induced injury	Cell-based (MSC)	Vascular (pulmonary artery)	Randomised preclinical transplant study, n = 12	Control without MSCs	Primary viability; IRI markers; inflammation; cell behaviour; post-transplant	Reduced apoptosis, oedema, and TNF-alpha with MSC treatment
	Nykänen 2021 [52]	Human	Mixed DCD/DBD declined donor lungs	Cell-based (MSC-IL-10)	Vascular (pulmonary artery)	Paired randomised ex vivo study, n = 9	Contralateral lung; wild-type MSC comparator	Primary viability; IRI markers; inflammation; cell behaviour	Confirmed transgene delivery feasibility; efficient IL-10 elevation; no improved physiological function
	Ribeiro 2025 [53]	Porcine/Human	DCD, 1 h no-touch warm ischemia	Cell-derived (exosomes)	Mixed/ multi-route	Controlled preclinical, n = 15 (3 arms)	NaCl Sham	Primary viability; IRI markers; inflammation; cell behaviour	Improved lung function, reduced injury and inflammatory markers with pre-DCD exosome delivery
	Stone 2017 [54]	Mouse	DCD, 60 min no-touch warm ischemia	Cell-based + cell-derived (MSC and EV)	Perfusate/circuit	Randomised preclinical, n = 5–10/arm (4 arms)	Steen solution alone; KH buffer; Sham; untreated IR	Primary viability; IRI markers; inflammation; cell behaviour	Reduced lung dysfunction and oedema with MSC/EV supplementation of EVLP
Heart	Zhang 2024 [55]	Rat/ Human (xeno)	DCD heart model, 15 min WIT	Cell-derived (exosomes)	Perfusate/circuit	Randomised preclinical transplantation study, n = 32	PBS Vehicle	Primary viability; IRI markers; inflammation; cell behaviour; post-transplant	Improved cardiac function, reduced apoptosis/inflammation, and better post-transplant recovery with exosome treatment

**Table 3:** Transplant-phase sensitivity analysis.

Organ	Study	Model	Follow-Up	Ex Vivo Signal	Post-Transplant Finding	Post-Transplant Outcome
**Kidney**	Lohmann 2021 [24]	Porcine auto-transplant	14 days	Reduced injury markers	GFR: no significant MSC-specific benefit reported (no stated *p*-value for this comparison). Plasma creatinine significantly lower with human MSCs, days 4–7 (*p* ≤ 0.032). One graft loss (NMP + vehicle, day 6, delayed graft function).	Mixed
	Garreta 2025 [32]	Porcine organoid auto-transplant	24–48 h	Confirmed engraftment; no injury model tested	Inflammatory cell infiltration (CD68+, CD15+, CD45+ tissue area): non-significant across all comparisons at 24 h and 48 h.	Mixed
**Liver**	Cao 2021 [37]	Rat allogeneic OLT	≥60 days	Reduced graft cytokines/rejection markers	Median survival >60 days (HBP), with survival statistically indistinguishable from FK506. BP group median survival reported inconsistently within the source paper: 21.5 days (results text) vs. 24.5 days (Figure 2 legend).	Beneficial
	Wu 2022 [43]	Rat allogeneic OLT	≥60 days	Inhibited DC maturation	Median survival >60 days (HBP). Rejection activity index (RAI) significantly worse for HBP vs. FK506 (*p* < 0.05), despite comparable survival times.	Mixed
	Tian 2021 [42]	Rat OLT	≥60 days	Regenerative/progenitor activation	Median survival >60 days (HBP); no FK506 or SCS comparator arm was included.	Beneficial *
**Lung**	Nakajima 2019 [51]	Porcine left lung transplant	4 h post-transplant	Molecular/histological improvement	Post-transplant tissue TNF-α (*p* = 0.03), wet/dry ratio (*p* = 0.02), and histologic lung injury score (*p* = 0.03) significantly improved; physiological function (PaO_2_/FiO_2_, compliance, peak airway pressure) unchanged.	Mixed
	Edström 2024 [46]	Porcine allotransplant (ARDS donor model)	3 days	No significant HGF induction	Zero primary graft dysfunction (PGD) events vs. significantly higher PGD in controls (*p* = 0.0041); PaO_2_/FiO_2_ and compliance significantly improved (*p* = 0.0043 each).	Beneficial
**Heart**	Zhang 2024 [55]	Rat heterotopic transplant	90 min	Reduced injury/apoptosis markers	Left ventricular developed pressure and dP/dt significantly improved at all three tested balloon volumes; dP/dt max significant at two of three (0.05, 0.10 mL), not significant at 0.15 mL—8 of 9 sub-comparisons significant overall.	Beneficial

* No SCS or FK506 comparator arm; benefit demonstrated only relative to NMP alone and BMMSC-alone controls.

## Data Availability

No new data were generated in this study. The data presented are available from the corresponding author upon reasonable request.

## Supplementary Materials

The following supporting information can be downloaded at: https://www.mdpi.com/article/10.3390/jcm15197698/s1, File S1: PRISMA 2020 Checklist [66]; File S2: Search Strategy.

## Abbreviations

The following abbreviations are used in this manuscript:

NMP	Normothermic Machine Perfusion
IRI	Ischaemia–Reperfusion Injury
SCS	Static Cold Storage
ECD	Extended Criteria Donor
DCD	Donation after Circulatory Death
HMP	Hypothermic Machine Perfusion
MSC	Mesenchymal Stromal Cell
EV	Extracellular Vesicle
MAPC	Multipotent Adult Progenitor Cell
