# Supplementary material for: Cell-Based and Cell-Derived Therapies Delivered During Ex Vivo Machine Perfusion for Solid Organ Reconditioning Prior to Transplantation: A Systematic Review
Source: J Clin Med. 2026 Oct 5;15(19):7698. doi: 10.3390/jcm15197698 (PMC13666490; doi:10.3390/jcm15197698)
Supplement: Supplementary file 1 [file jcm-15-07698-s001.zip › jcm-4592278-File S2.pdf]

Supplementary Material

Appendix A

Search Strategy

((("machine perfusion"[tiab] OR "normothermic perfusion"[tiab] OR "hypothermic perfusion"[tiab] OR EVNP[tiab] OR EVLP[tiab] OR NMP[tiab] OR HMP[tiab] OR HOPE[tiab] OR "organ care system"[tiab])

AND

(mesenchymal[tiab] OR MSC[tiab] OR "stem cell*"[tiab] OR "stromal cell*"[tiab] OR organoid*[tiab] OR vesicle*[tiab] OR exosome*[tiab] OR progenitor*[tiab] OR MAPC[tiab]))

AND

(transplant*[tiab] OR graft*[tiab] OR donor*[tiab] OR kidney*[tiab] OR liver*[tiab] OR lung*[tiab] OR heart*[tiab]))
